# Therapeutic effects of repetitive transcranial magnetic stimulation on cognitive impairment in stroke patients: a systematic review and meta-analysis

**DOI:** 10.3389/fnhum.2023.1177594

**Published:** 2023-05-12

**Authors:** Cheng Gong, Hao Hu, Xu-Miao Peng, Hai Li, Li Xiao, Zhen Liu, Yan-Biao Zhong, Mao-Yuan Wang, Yun Luo

**Affiliations:** ^1^Gannan Medical University, Ganzhou, Jiangxi, China; ^2^Department of Rehabilitation Medicine, First Affiliated Hospital of Gannan Medical University, Ganzhou, Jiangxi, China; ^3^Ganzhou Intelligent Rehabilitation Technology Innovation Center, Ganzhou, Jiangxi, China; ^4^Ganzhou Key Laboratory of Rehabilitation Medicine, Ganzhou, Jiangxi, China

**Keywords:** stroke, cognitive impairment, repetitive transcranial electrical stimulation, systematic review, meta-analysis

## Abstract

**Background:**

In recent years, repetitive transcranial magnetic stimulation (rTMS) has emerged as a noninvasive and painless treatment for post-stroke cognitive impairment (PSCI). However, few studies have analyzed the intervention parameters of cognitive function and the effectiveness and safety of rTMS for treating patients with PSCI. Thus, this meta-analysis aimed to analyze the interventional parameters of rTMS and evaluate the safety and effectiveness of rTMS for treating patients with PSCI.

**Methods:**

According to the PRISMA guidelines, we searched the Web of Science, PubMed, EBSCO, Cochrane Library, PEDro, and Embase to retrieve randomized controlled trials (RCTs) of rTMS for the treatment of patients with PSCI. Studies were screened according to the inclusion and exclusion criteria, and two reviewers independently performed literature screening, data extraction, and quality assessment. RevMan 5.40 software was used for data analysis.

**Results:**

12 RCTs involving 497 patients with PSCI met the inclusion criteria. In our analysis, rTMS had a positive therapeutic effect on cognitive rehabilitation in patients with PSCI (*P* < 0.05). Both high-frequency rTMS and low-frequency rTMS were effective in improving the cognitive function of patients with PSCI by stimulating the dorsolateral prefrontal cortex (DLPFC), but their efficacy was not statistically different (*P* > 0.05).

**Conclusions:**

rTMS treatment on the DLPFC can improve cognitive function in patients with PSCI. There is no significant difference in the treatment effect of high-frequency rTMS and low-frequency rTMS in patients with PSCI between high-frequency and low-frequency rTMS.

**Systematic review registration:**

https://www.crd.york.ac.uk/prospero/display_record.php?RecordID=323720, identifier CRD 42022323720.

## Introduction

Post-stroke cognitive impairment (PSCI) refers to a type of cognitive impairment caused by stroke with cerebrovascular insults and neurodegenerative changes, resulting in progressively decreased function in attention, memory, execution, and other cognitive domains, which can progress further to dementia (Mijajlović et al., 2017). PSCI is the leading cause of massive vascular cognitive impairment syndrome, including post-stroke mild neurocognitive disorder that do not fulfill the criteria for dementia and post-stroke dementia (PSD) (Mijajlović et al., 2017). Previous studies have reported the prevalence of PSCI to be 24–53.4% (Douiri et al., 2013; Lo et al., 2019), with the prevalence of post-stroke dementia (PSD) ranging from 11 to 42% and post-stroke mild neurocognitive disorder ranging from 14 to 29% (Munthe-Kaas et al., 2020). Patients with PSCI experienced a significantly higher mortality rate than those without. According to the researchers, the 5-year survival rate for patients with PSD is only 39% compared to 75% for stroke patients of the same age without dementia (Danovska et al., 2012). PSCI is associated with increased disability, dependency, and mobility, posing a major burden to patients, caregivers, and healthcare systems (Rohde et al., 2019; Huang et al., 2022).

The main current approaches to treating PSCI include pharmacotherapy and cognitive rehabilitation. Pharmacological treatment with cholinesterase inhibitors (donepezil, galantamine, etc.) improves patients' cognitive function and daily living activities (Wilkinson et al., 2003; Craig and Birks, 2006). However, many studies have shown that drug-only treatment usually suffers from unsatisfactory efficacy, inconsistent patient tolerance and excessive negative effects, which is not recommended as a priority treatment in practice guidelines (Crane and Doody, 2009; Tricco et al., 2013; Fitzpatrick-Lewis et al., 2015; Petersen et al., 2018). Cognitive rehabilitation training is a common rehabilitation treatment to promote the cognitive recovery of patients. However, cognitive rehabilitation training has poor patient cooperation, prolonged time, and effort, resulting in a slow treatment effect.

In recent years, transcranial magnetic stimulation (TMS) has been widely used in the treatment of neuromodulation of the brain because of its efficacy in modulating the activity of cortical areas (Mehta et al., 2019; Blech and Starling, 2020; Chung et al., 2020; Mimura et al., 2021). The therapeutic mechanism of TMS is to generate subthreshold or suprathreshold currents in the human cerebral cortex through electromagnetic induction (Lefaucheur et al., 2014; Rossini et al., 2015), thereby modulating the excitability of the cerebral cortex. According to the stimulation frequency, TMS can be divided into high-frequency (>1 Hz) TMS and low-frequency ( ≤ 1 Hz) TMS. Studies have shown that high-frequency TMS can increase the excitability of the cerebral cortex in the stimulated area, while low-frequency TMS can inhibit the excitability of the cerebral cortex (Fitzgerald et al., 2006). TMS can be divided into single-pulse, double-pulse and repetitive transcranial magnetic stimulation (rTMS), depending on the stimulation pulse. And rTMS also includes two special stimulation modes: intermittent theta burst stimulation (iTBS) and continuous theta burst stimulation (cTBS). Among them, rTMS has been proposed as an emerging non-invasive and painless treatment to improve patients' cognitive function (Magavi et al., 2017). In recent years, many researchers are trying to improve the cognitive function of patients with PSCI by modulating the excitability of the cortical areas responsible for cognitive control through rTMS (Randver, 2018).

However, the role of rTMS in PSCI remains unclear. Some studies found (Liberati et al., 2009; Randver, 2018; Liu et al., 2021; Li et al., 2023) that rTMS improved the cognitive function of patients with PSCI, while others showed that rTMS did not improve the cognitive function in patients with PSCI (Sterne et al., 2019). The first systematic review and meta-analysis with a small sample size was published in 2021 (Higgins et al., 2003) and found that rTMS have a positive effect on improving the cognitive ability of stroke patients. Recently, six new RCTs (Guyatt et al., 2011; Mercuri et al., 2018; Cumpston et al., 2019; Yin et al., 2020; Li et al., 2021, 2022; Chu et al., 2022) have been published. As a result, an updated systematic review and meta-analysis is needed to provide high-quality evidence about the effect of rTMS on PSCI. At the time of writing our manuscript, a new systematic review and meta-analysis (Tsai et al., 2020) has been published. However, we have a theoretical concern on the data analysis of that study because the authors only extracted the post-intervention data and ignored the pre-intervention difference, which might yield an unreliable conclusion. Thus, in this meta-analysis, we included the latest randomized controlled trials (RCTs) to analyze the interventional parameters of rTMS and evaluate the effectiveness and safety of rTMS for treating patients with PSCI.

## Methods

### Protocol and registration

Our systematic review was designed and implemented based on the Preferred Reporting Items for Systematic Reviews and Meta-analysis (PRISMA) guideline (Liberati et al., 2009). The study has been registered with Prospero (CRD 42022323720).

### Retrieval strategy

Searches were made in the below databases on Web of Science, PubMed, EBSCO, Cochrane library, PEDro, and Embase, from the initial availability date to 6 April 2023, to identify articles to be included in the quantitative analysis. The main search terms for this study were “Stroke,” “Cognitive impairment,” “Attention,” “Memory,” “Executive Function,” and “Repetitive Transcranial Magnetic Stimulation.” In addition, we manually searched other relevant literature, such as studies included in some systematic reviews and meta-analyses, to broaden the search for eligible articles. For instance, the following search strategy was used for PubMed: ((Stroke) OR (Cerebrovascular Accident) OR (Brain Vascular Accident) OR (Cerebrovascular Accidents)) AND (Cognitive impairment) OR (Cognitive dysfunction) OR (Cognitive Function) OR (Cognitive Functions) OR (Attention) OR (Memory) OR (Executive Function)) AND ((Repetitive Transcranial Magnetic Stimulation) OR (Transcranial Magnetic Stimulation) OR (TMS) OR (rTMS) OR (iTBS)). A similar search strategy was used for the other databases.

### Inclusion and exclusion criteria

Inclusion and exclusion criteria for literature screening were predetermined to allow for more rigorous literature screening. Inclusion criteria of the literature: (1) patients diagnosed with PSCI by clinical examination; (2) trial group received rTMS treatment intervention with cognitive function training; (3) control group received sham rTMS treatment or cognitive function training only; (4) outcome indicators of cognitive function were assessed by relevant scales, such as the Mini-Mental State Examination (MMSE), Loewenstein Occupational Therapy's Cognitive Assessment (LOTCA), Montreal Cognitive Assessment (MoCA); (5) The experimental design was a randomized controlled trial;(6) the language was restricted to English.

Exclusion criteria of the literature: (1) animal experiments; (2) cognitive impairment due to other etiologies (e.g., cranial trauma, Alzheimer's disease, Parkinson's disease, etc.); (3) experimental groups received interventions other than rTMS with cognitive function training; (4) full-text content was not available; (5) experimental data were missing or duplicated.

### Study selection

We imported all retrieved studies into the document management system of Endnote 20 and removed duplicate studies using software management features. Two researchers (CG and HH) perused the title and abstract of each study simultaneously and then screened studies based on the criteria we had previously developed. For further screening, two reviewers downloaded and viewed the full text to remove articles that did not meet the inclusion criteria and to confirm their eligibility. If there was disagreement in the screening process, a consensus was reached through the advice provided by the principal investigator (YL).

### Data extraction

Two reviewers (CG and HH) independently extracted the following data and items from the included literature: the first author of the study, year of publication, sample size, age of patients, gender, disease duration, type of stroke, stroke site, interventions, and outcome indicators of the trial. Specific stimulation parameters, including stimulation site, frequency, intensity, and the number of pulses per unit, were also recorded in detail for the intervention method. In addition, when two reviewers (CG and HH) encountered unclear or complicated extraction of complete literature data during data extraction, the original authors of the literature would be contacted to obtain the complete experimental data. After three consecutive emails are sent, the study will be defined as missing data if no response is received from the original author.

### Quality assessment and risk of bias assessment

Quality assessment of the literature studies was completed independently by two researchers (CG and HH), followed by a discussion to produce consistent results. Risk bias assessment was conducted using the Cochrane Risk of Bias tool (Review Manager 5.40). Items were assessed from seven areas: blinding of participants and personnel, blinding of outcome assessments, allocation concealment, random sequence generation, incomplete outcome data, selective reporting, and other biases. Each risk bias was graded as high risk, low risk, or unclear (Sterne et al., 2019). The assessment of risk bias in each study's re-risk bias assessment item was recorded by mapping the risk bias assessment.

Heterogeneity between studies was statistically analyzed by Revman 5.40. The size of heterogeneity was expressed as *I*^2^, and heterogeneity was judged as high heterogeneity when *I*^2^ ≥ 75%, moderate heterogeneity when 75% > *I*^2^ ≥ 50%, low heterogeneity when 50% > *I*^2^ ≥ 25%, and no heterogeneous heterogeneity if *I*^2^ = 0% (Higgins et al., 2003).

The quality of evidence for outcome indicators was assessed using the Grading of Recommendations Assessment, Development, and Evaluation (GRADE) system, which examines areas, such as study limitations, intermittency, inconsistency, and imprecision of results (Guyatt et al., 2011; Mercuri et al., 2018). The results were assessed by grading the evidence for the outcome indicators as “high,” “moderate,” “low,” or “very low,” and the strength of the recommendations was divided into two levels: strong and weak (Guyatt et al., 2011).

### Statistical analysis

For statistical and analytical purposes, extracted study data were entered into Revman 5.40 software. Depending on the magnitude of heterogeneity, the decision was made to use a fixed-effects model or a random-effects model for the meta-analysis. When *I*^2^ ≥ 50%, a random effects model was used; when *I*^2^ < 50%, a fixed effects model was used (Cumpston et al., 2019). We report 95% confidence intervals (CI) and mean difference (MD) to express the effect size. The effect size of different units of the same outcome index was measured by standardized mean difference (SMD).

## Results

### Research result

**Figure 1:**
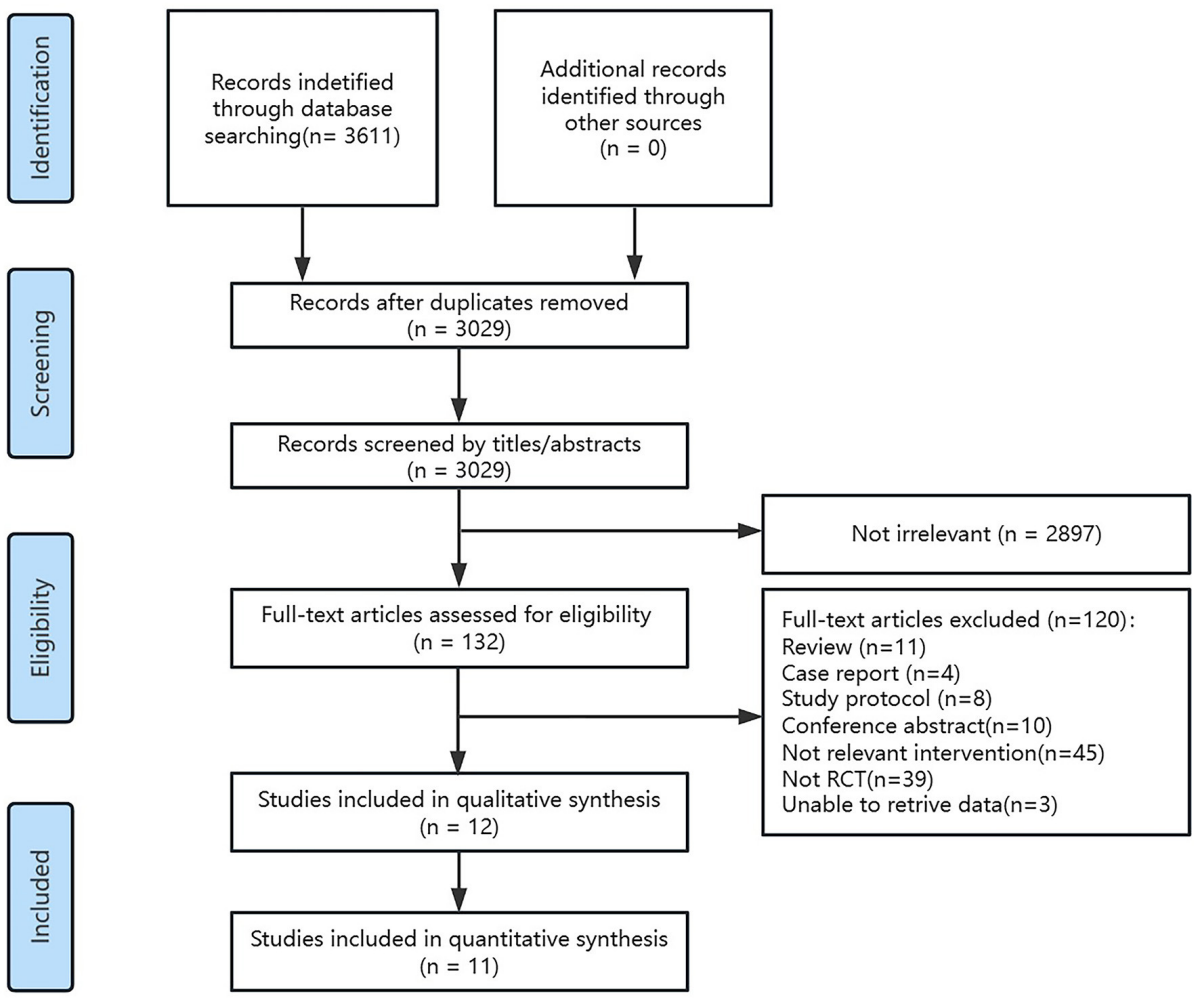
Flow graph of selection and exclusion.

Relevant literatures were searched from the six databases and the initial search yielded 3,611 studies. After screening for duplicate studies using the software deletion function, 3,029 studies remained. Then, two reviewers read the titles and abstracts of these studies and screened out 2,897 irrelevant to the topic. The remaining 132 studies were assessed by full text and 120 studies excluded, including 11 reviews, 4 case reports, 8 study protocols, 10 conference abstracts, 45 studies with no relevant interventions, 39 non-randomized controlled trial studies, and 3 studies unable to retrive with no data. Ultimately, 12 eligible studies were included in this study (Du and Wu, 2005; Fregni et al., 2006; Kim et al., 2010; Lu et al., 2015; Liu, 2017; Liu et al., 2020; Li et al., 2020, 2021, 2022; Tsai et al., 2020; Yin et al., 2020; Chu et al., 2022). One study (Tsai et al., 2020) did not use any of the same outcome indicators as the rest studies, so only 11 studies were included for quantitative analysis (Figure 1).

### Characteristics of the trials

Clinical characteristics and interventional parameters were extracted of the 12 included RCTs are summarized in Tables 1, 2, respectively. A total of 497 patients eligible for the study were included, with sample sizes ranging from 15 to 65 per RCT, with differences in the hemisphere of stroke site, type, and duration. The number of PSCI patients treated with rTMS was 258, with an additional 239 receiving placebo or cognitive function training only. The intervention characteristics of rTMS treatment, such as stimulation site, intensity, frequency, number of pulses, and duration, are excerpted in Table 2. In our meta-analysis, five studies (Du and Wu, 2005; Fregni et al., 2006; Kim et al., 2010; Lu et al., 2015; Li et al., 2021) with a total of 112 patients received low-frequency transcranial magnetic stimulation, and 8 studies (Kim et al., 2010; Liu, 2017; Liu et al., 2020; Li et al., 2020, 2022; Tsai et al., 2020; Yin et al., 2020; Chu et al., 2022) with a total of 159 patients received high-frequency transcranial magnetic stimulation.

**Table 1:** The main characteristics of the reviewed studies.

**References**	**Sample size (*n*)**	**Sex (M/F)**	**Age (years), M ±SD**	**Type of stroke (hemorrhagic/ ischemic)**	**stroke site (left/ right/ bilateral)**	**Disease duration**	**Outcome**	**Adverse events**
1. Du and Wu (2005)	G1:30 G2:30	34/26	57.6 ± 10.8	–	15/10/5 16/10/4	–	MMSE MBI	No
2. Kim et al. (2010)	G1:6 G2:6 G3:6	2/4 4/2 4/2	68.3 ± 7.4 53.5 ± 16.9 66.8 ± 17.2	1/5 2/4 1/5	–	404.4 ± 71.7days 241.2 ± 42.5day 69.7 ± 39.0days	MBI	No
3. Lu et al. (2015)	G1:19 G2:21	12/7 13/8	42.5 ± 12.3 47.3 ± 11.8	11/8 11/10	1/8/0 1/10/0	67 ± 30days 56 ± 30days	LOTCA MoCA	Dizziness
4. Liu (2017)	G1:18 G2:18	11/7 9/9	65.33 ± 7.05 62.61 ± 9.98	13/5 12/6	–	253.2 ± 53.7 days 279.9 ± 49.2 days	MMSE	No
5. Liu et al. (2020)	G1:29 G2:29	10/19 16/13	58.6 ± 6.2 57.7 ± 7.3	9/20 15/14	11/18/0 14/15/0	8.7 ± 1.8 months 8.6 ± 1.8 months	MMSE	NR
6. Yin et al. (2020)	G1:16 G2:18	14/2 16/2	56.7 ± 12.9 58.2 ± 11.3	5/11 6/12	4/6/6 6/7/5	52 ± 38.3 days 55 ± 39.5 days	MoCA	No
7. Li et al. (2020)	G1:15 G2:15	7/8 9/6	65.5 ± 3.7 64.5 ± 4.7	–	5/10/0 6/9/0	22.7 ± 8.1days 19.1 ± 7.9 days	MMSE MoCA	Dizziness Headaches
8. Tsai et al. (2020)	m G1:11 G2:15 G3:15	9/2 11/4 13/2	57.5 ± 12.3 60.1 ± 14.1 56.2 ± 12.0	3/8 7/8 10/5	11/0/0 15/0/0 15/0/0	33.3 ± 26.4 months 18.8 ± 20.2 months 38 ± 7.9 months	RBANS	No
9. Li et al. (2021)	G1:33 G2:32	12/21 19/13	61.8 ± 5.5 59.5 ± 6.6	22/11 18/14	15/18/0 13/19/0	28.6 ± 12.6 days 27.8 ± 11.0 days	MoCA MBI	No
10. Fregni et al. (2006)	G1:10 G2:5	8/2 3/2	57.70 ± 11.2752.60 ± 12.56	–	8/2/0 4/1/0	105.6 ± 87.9 days 119.1 ± 79.2 days	MMSE	Anxiety Tiredness mild headache
11. Chu et al. (2022)	G1: 21 G2: 20	18/3 13/7	57.24 ± 14.03 66.75 ± 12.23	8/13 8/12	12/9/0 13/7/0	4.00 ± 5.00 months 6.00 ± 4.00 months	MBI LOTCA	No
12. Li et al. (2022)	G1:28 G2:30	16/12 18/12	69.5 ± 12.7 66.0 ± 15.6	10/18 16/14	12/16/0 6/24/0	25 ± 9.19 days 25 ± 8.49 days	MMSE	Sneezing

G1, group 1; G2, group 2; G3, group 3; n, number; M, male; F, female; LF, low frequency; “–”, not given; NR, not reported, MMSE, Mini-Mental Status Examination; MBI, Modifed Barthel Index; MoCA, Montreal Cognitive Assessment; RBANS, Repeatable Battery for the Assessment of Neuropsyehological Status.

**Table 2:** Main intervention parameters of rTMS.

**References**	**Intervention method**	**Stimulation position**	**Intensity (%MT)**	**Frequency (Hz)**	**Total pulses**	**Number of sessions (day)**	**Duration of intervention**	**Cognitive training**
1. Du and Wu (2005)	G1: rTMS G2: sham-rTMS	DLPFC (Bilateral)	60	0.5	100	20	4w, 5d/w	Yes
2. Kim et al. (2010)	G1: rTMS G2: rTMS G3: sham-rTMS	L-DLPFC (unilateral)	LF: 80 HF: 90	LF: 1 HF: 10	LF: 900 HF: 450	10	2w, 5d/w	Yes
3. Lu et al. (2015)	G1: rTMS G2: sham-rTMS	R-DLPFC (Unilateral)	100	1	600	20	4w, 5d/w	Yes
4. Liu (2017)	G1: rTMS G2: sham-rTMS	L-DLPFC (unilateral)	100	10	700	20	4w, 5d/w	Yes
5. Liu et al. (2020)	G1: rTMS G2: sham-rTMS	L-DLPFC (Unilateral)	100	10	700	20	4w, 5d/w	Yes
6. Yin et al. (2020)	G1: rTMS G2: sham-rTMS	L-DLPFC (Unilateral)	60	10	2,000	20	4w, 5d/w	Yes
7. Li et al. (2020)	G1: rTMS G2: sham-rTMS	L-DLPFC (Unilateral)	90	5	2,000	15	3w, 5d/w	Yes
8. Tsai et al. (2020)	G1: rTMS G2: iTBS G2: sham-rTMS	L-DLPFC (Unilateral)	90	5 50/5 5	600	10	2w, 5d/w	Yes
9. Li et al. (2021)	G1: rTMS G2: sham-rTMS	DLPFC (contralateral)	90	1	1,000	20	4w, 5d/w	Yes
10. Fregni et al. (2006)	G1: rTMS G2: sham-rTMS	M1	100	1	1,200	5	1w, 5d/w	Yes
11. Chu et al. (2022)	G1: iTBS+ Cognitive training G2: Cognitive training	L-DLPFC (unilateral)	70	50/5	600	30	6w, 5d/w	Yes
12. Li et al. (2022)	G1: iTBS G2: sham-iTBS	L-DLPFC (unilateral)	100	50/5	600	10	2w, 5d/w	Yes

LF, low frequency; HF, high frequency; R-DLPFC, the right side of the dorsolateral prefrontal cortex; L-DLPFC, the left prefrontal cortex; DLPFC, the dorsolateral prefrontal cortex; M1, primary motor cortex M1 area.

### Quality assessment result

**Figure 2:**
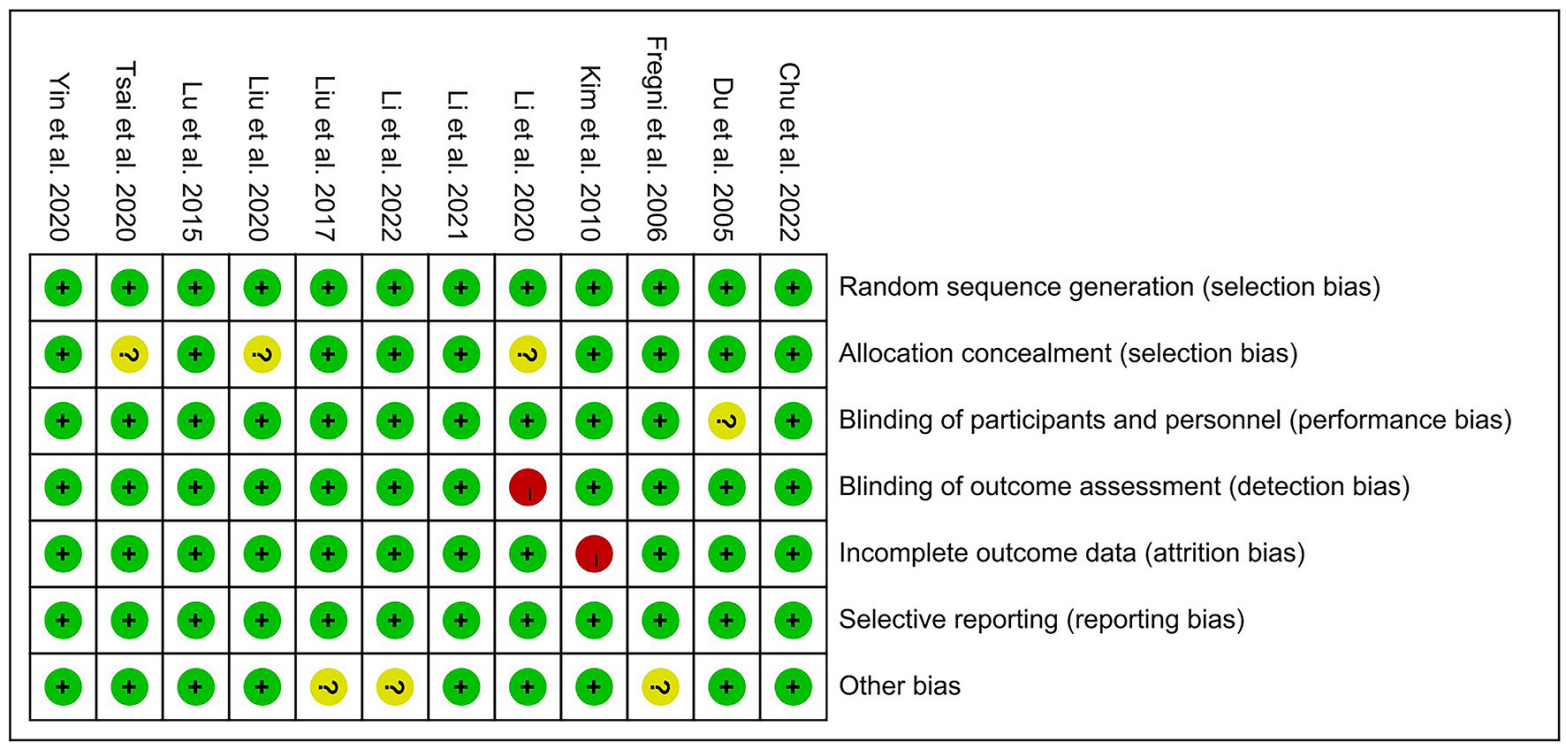
Risk of bias graph: judgements about each risk of bias item presented as percentages.

**Figure 3:**
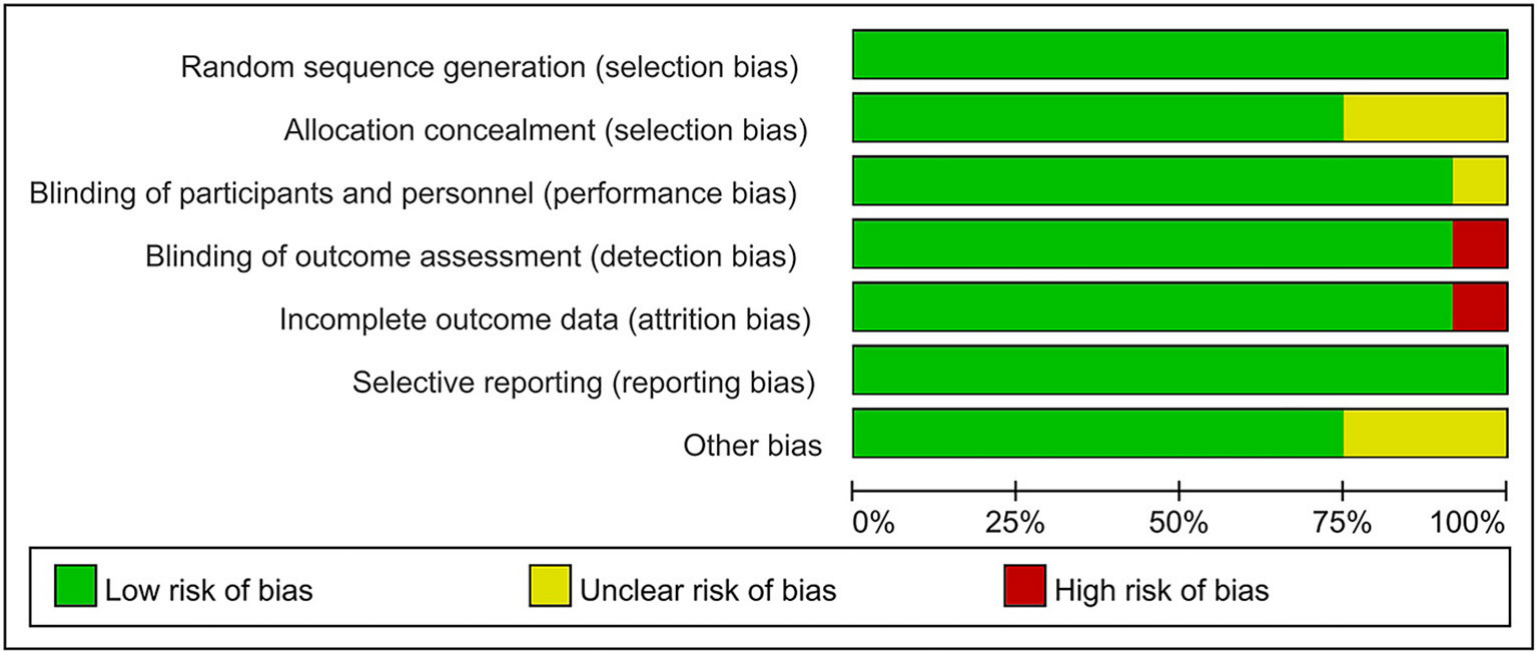
Risk of bias summary: judgements about each risk of bias item for each included study.

The risk of bias of the 12 included RCTs were assessed according to the Cochrane Risk of Bias Assessment Tool (Revman 5.40). One studies (Kim et al., 2010) failed to give data variation for all assessments in the reporting of study results and had a high risk of attrition bias. One study (Li et al., 2020) was not reasonably blinded in the assessment of outcome indicators, leaving it with a high risk. None of the rest studies had high-risk bias items in the assessment of bias risk. Overall, the risk of bias in our included RCTs was low (Figures 2, 3).

We evaluated the GRADE evidence level recommendations for the main outcome indicators of MMSE, MoCA, LOTCA, and MBI (Modified Barthel Index) (Table 3). Due to the small sample sizes involved in the outcome indicators of MMSE, MoCA, and LOTCA (*n* < 200), we rated the risk of imprecision for these 3 outcome indicators as “severe” with an intermediate level of evidence. Because of the small sample size (*n* < 200) and high heterogeneity (*I*^2^ > 80%) of the studies with MBI as an outcome indicator, the risk of imprecision and Inconsistency for MBI outcome indicators were both rated as “severe” with a low level of evidence. However, no serious risks were identified in the rest of the items assessed, and therefore the overall GRADE evidence recommendation for this study was “strong.”

**Table 3:** Grading of Recommendations Assessment, Development, and Evaluation (GRADE) quality of evidence.

**Outcome**	**Number of studies**	**Design**	**Study limitations**	**Inconsistency**	**Indirectness**	**Imprecision**	**Publication bias**	**Effect size**	**GRADE quality**	**Symbolic expression**
MMSE	6	RCT	0	0	0	−1^#^	0	0	Moderate	⊕⊕⊕⊖
MoCA	4	RCT	0	0	0	−1^#^	0	0	Moderate	⊕⊕⊕⊖
LOTCA	2	RCT	0	0	0	−1^#^	0	0	Moderate	⊕⊕⊕⊖
MBI	4	RCT	0	−1^*^	0	−1^#^	0	0	Low	⊕⊕⊖⊖

MMSE, Mini-Mental Status Examination; MoCA, Montreal Cognitive Assessment; LOTCA, Loewenstein Occupational Therapy's Cognitive Assessment; MBI, Modified Barthel Index.

The symbol “⊕⊕⊕⊖” means the level of evidence is graded as moderate.

The symbol “⊕⊕⊖⊖” means the level of evidence is graded as low.

The number of the symbol “⊕” represents the level of evidence; the higher the number, the higher the level.

⊕⊕⊕⊕: High evidence level; ⊕⊕⊕⊖: Moderate evidence level; ⊕⊕⊖⊖: Low evidence level; ⊕⊖⊖⊖: Very Low evidence level.

^#^n < 200.

^*^I^2^ > 80%.

### Results of statistical analysis

**Figure 4:**
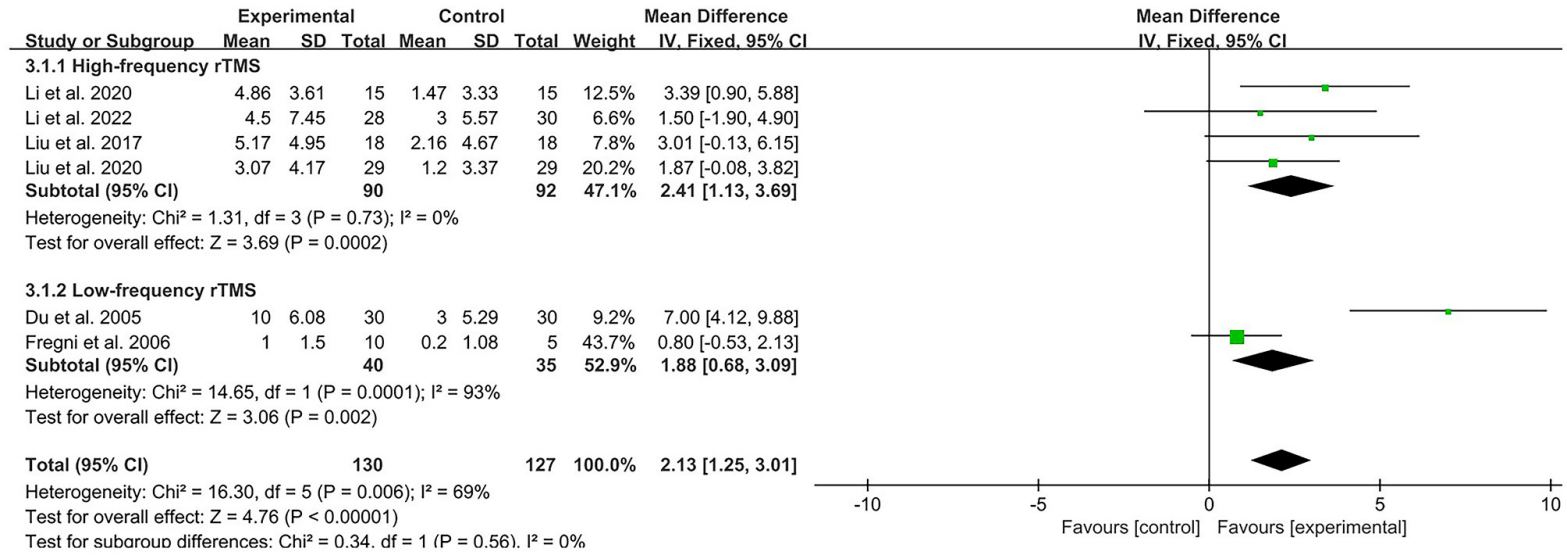
Forest plot analysis of rTMS based on MMSE scores.

Six studies (Du and Wu, 2005; Fregni et al., 2006; Liu, 2017; Liu et al., 2020; Li et al., 2020, 2022) used MMSE to assess the cognitive function of patients with PSCI by calculating the heterogeneity between studies with *I*^2^ < 50%, therefore a fixed effects model was used for data analysis. Of them, four studies were treated with high-frequency rTMS and two studies were treated with low-frequency rTMS. Compared to controls, high-frequency rTMS stimulation of patients' dorsolateral prefrontal cortex (DLPFC) showed significant improvement in improving MMSE scores in patients with PSCI [MD = 2.41, 95% CI (1.13, 3.69), *I*^2^ = 0%, *P* < 0.05, Figure 4], and low-frequency rTMS stimulation of patients' DLPFC similarly promoted cognitive function in patients with PSCI [MD = 1.88, 95% CI (0.68, 3.09), *I*^2^ = 93%, *P* < 0.05, Figure 4] treatment improved the cognitive function of patients with PSCI, respectively. In addition, there was no statistical difference in the efficacy of the two frequencies of rTMS (*P* > 0.05).

**Figure 5:**
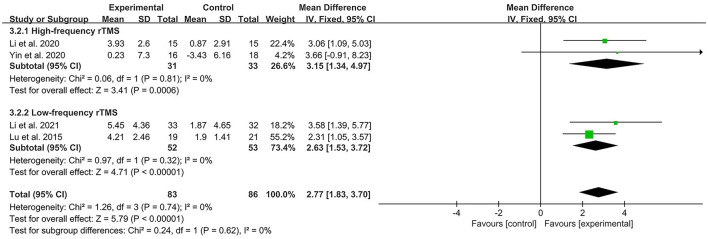
Forest plot analysis of rTMS based on MoCA scores.

Four studies (Lu et al., 2015; Li et al., 2020, 2021; Yin et al., 2020) rated the cognitive function of patients by the MoCA rating scale, and by calculating the heterogeneity between studies, *I*^2^ < 50%, so a fixed-effects model was used for data analysis. Compared to controls, DLPFC in patients with high-frequency rTMS stimulation was effective in improving cognitive function in patients with PPSCI [MD = 3.15, 95% CI (1.34, 4.97), *I*^2^ = 0%, *P* < 0.05, Figure 5], and DLPFC in patients with low-frequency rTMS stimulation was equally effective in enhancing cognition in patients with PPSCI [MD = 2.63, 95% CI (1.53, 3.72), *I*^2^ = 0%, *P* < 0.05, Figure 5]. In addition, subgroup analysis showed no statistical difference in the efficacy of rTMS between the two frequencies (*P* > 0.05).

**Figure 6:**
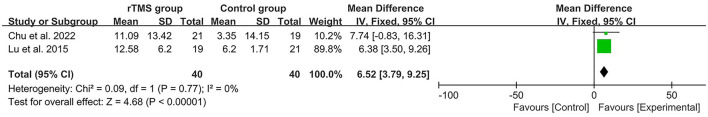
Forest plot analysis of rTMS based on LOTCA scores.

Two studies (Lu et al., 2015; Chu et al., 2022) rated the degree of loss of cognitive function in patients using the LOTCA scale. And by calculating the heterogeneity between studies, *I*^2^ < 50%, so a fixed-effects model was used for data analysis. Data analysis of the forest plot showed that rTMS stimulation of patients' DLPFC significantly improved LOTCA scores in patients with PSCI compared with controls [MD = 6.52, 95% CI (3.79, 9.52), *I*^2^ = 0%, *P* < 0.05, Figure 6].

**Figure 7:**
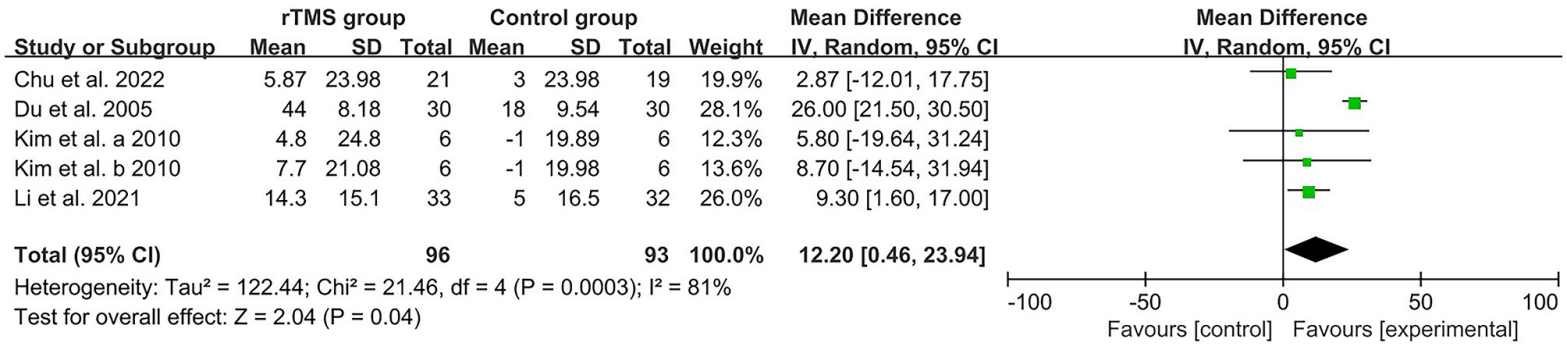
Forest plot analysis of rTMS based on MBI scores.

Four studies (Du and Wu, 2005; Kim et al., 2010; Li et al., 2021; Chu et al., 2022) used the MBI scale to assess the ability of patients with PSCI to perform activities of daily living, and by calculating the heterogeneity between studies, *I*^2^ > 50%, therefore a random effects model was used for data analysis. The results of the forest plot analysis showed that rTMS stimulation of patients' DLPFC was effective in improving patients' activities of daily living compared with controls [MD = 12.2, 95% CI (0.46, 23.94), *I*^2^ = 81%, *P* < 0.05, Figure 7].

### Adverse event reporting results

Among the included studies, 11 studies (Du and Wu, 2005; Fregni et al., 2006; Kim et al., 2010; Lu et al., 2015; Liu, 2017; Li et al., 2020, 2021, 2022; Tsai et al., 2020; Yin et al., 2020; Chu et al., 2022) documented adverse effects when rTMS was administered to patients with PSCI, but only three studies reported the occurrence of adverse events. Four studies (Fregni et al., 2006; Lu et al., 2015; Li et al., 2020, 2022) reported the presence of transient vertigo and headache after patients received rTMS, and 1 study (Li et al., 2022) reported that patients induced sneezing when receiving rTMS. Based on the results reported in all studies, no patients withdrew from the experimental studies because they exhibited excessive adverse effects.

## Discussion

### Main meta-analysis results

Both low-frequency and high-frequency rTMS on DLPFC were effective in improving cognitive function and enhancing the ability of daily activities in patients with PSCI. The subgroup analysis showed that there was no significant difference between low-frequency and high-frequency rTMS in improving the cognitive function of patients.

### Comparison with previous studies

Compared to previous studies we included the most recent RCTs for meta-analysis. Moreover, our data analysis was performed on the amount of change in the rated indicators before and after the trial, not just on the values of the outcome indicators after the experimental intervention. We also supplemented the study of Liu et al. (2021) with an analysis of the effectiveness of rTMS in improving the activities of daily living in patients with PSCI. And compared to the Li et al. study (Li et al., 2023), we additionally added the data analysis of LOTCA score as an outcome indicator to improve the reliability of the overall analysis results. Finally, we also added the neglected safety analysis of rTMS for PSCI patients. In addition, our subgroup analysis of the difference in efficacy of rTMS at different frequencies in patients with PSCI yielded results inconsistent with the study of Li et al. Li et al. showed that low-frequency rTMS did not improve patients' MoCA scores compared with controls. However, our subgroup analysis showed that low-frequency rTMS improved the MoCA scores of patients and that there was no statistical difference in efficacy compared with high-frequency rTMS. In this regard, we have explored in more depth the theoretical mechanisms of rTMS in the treatment of patients with PSCI.

Studies indicate that rTMS can produce an electric field in the brain based on the principle of electromagnetic induction, which induces depolarized neurons to regulate cortical excitability (Thibaut et al., 2019). And the best application of rTMS as a neuromodulatory capability for different stroke patients is worth exploring in depth. The theoretical mechanisms of rTMS reported in published studies are mostly based on the “hemispheric competition model” (Rossi et al., 2021). In humans, both cerebral hemispheres are in a state of mutual inhibition equilibrium under normal physiological conditions, often referred to as “transcallosal mutual inhibition.” However, the cerebral hemispheric equilibrium of mutual inhibition can be disrupted in stroke patients with brain damage. For example, it was found that the dorsolateral prefrontal cortex (DLPFC) is the higher control area that controls cognition (Ma et al., 2022). When patients with damage to the left dorsolateral prefrontal area experience cognitive dysfunction, it leads to decreased inhibition of the right cerebral hemisphere. This situation further leads to the overactivation of the right cerebral hemisphere and increased inhibition of the left cerebral hemisphere by the right cerebral hemisphere, disrupting the balance of inhibition in both hemispheres and affecting the cognitive function recovery in patients with PSCI. It is vital to regulate the excitability of the cerebral cortex on both sides to correct the imbalance between the two cerebral hemispheres, and rTMS can achieve this. It has been found that high-frequency rTMS increases cortical excitability and low-frequency rTMS decreases cortical excitability (de Aguiar et al., 2015; Hara et al., 2015). Through this mechanism, rTMS can regulate the imbalance in both cerebral hemispheres (Julkunen et al., 2009; Takechi et al., 2014; Pastuszak et al., 2016) and foster plasticity changes in the cerebral cortex, thus promoting cognitive recovery in patients with PSCI. However, some researchers have found that the theoretical model of interhemispheric competition dominates in patients with less severe hemispheric damage, but is less relevant in patients with more severe hemispheric damage (Werhahn et al., 2003; Johansen-Berg, 2007; Seghier, 2008; Johansen-Berg et al., 2010). In light of these findings, Di Pino et al. (2014) proposed a bimodal balance-recovery model for the clinical use of non-invasive brain stimulation (NIBS) in stroke patients, which suggested that when treating stroke patients with rTMS, a high priority should be given to the “structural reserve” (the extent to which neural pathways and connections are preserved after stroke) of stroke patients. Di Pino et al. showed that a high structural reserve in stroke patients predicted recovery better with an interhemispheric competition model, and a low structural reserve predicted recovery better with a hemispheric compensatory model. The theoretical mechanism of the hemispheric compensation model is that when the hemispheric damage is too severe and the structural reserve is low, rTMS excitation of the patient's hemispheres is no longer sufficient to provide adequate functional compensation, but rather excitation of the healthy hemispheres should be increased to achieve a better treatment outcome by expanding the functional compensation of the healthy hemispheres. Nair et al. (2007) have also demonstrated the important role that the healthy hemisphere plays in the recovery of stroke patients.

Previous randomized controlled trials have reported different degrees of facilitation of cognitive function in PSCI patients by rTMS (Kim et al., 2010; Tsai et al., 2020), and these experimental studies used different frequencies of rTMS to treat PSCI patients. To clarify the effects of different rTMS frequencies on cognitive function in PSCI patients, we performed a subgroup analysis of the differences in the efficacy of g high-frequency rTMS and low-frequency rTMS. The results showed that both high and low frequencies of rTMS positively improved cognitive function in PSCI patients. There was no significant difference in the improvement of cognitive function between the two frequencies, which is consistent with the findings of Kim et al. (2010). However, it is noteworthy that Kim et al. found that high-frequency rTMS was more effective than low-frequency rTMS in reducing depression when they analyzed the treatment effects of different rTMS frequencies. Kim's study suggests to us that although there was no significant difference between low-frequency and high-frequency rTMS in improving the scores of cognitive function in patients with PSCI, the treatment effects were not exactly similar in treating depression. In this regard, we believe it is necessary to take into account the presence of depression, among other things, when selecting different frequencies of rTMS for the treatment of patients with PSCI.

There are three studies (Tsai et al., 2020; Chu et al., 2022; Li et al., 2022) among our included studies that used iTBS for the treatment of patients with PSCI, but because the outcome indicators were inconsistent among these three studies, we could only perform a systematic review and analysis. iTBS as a special stimulation mode of rTMS, has a shorter treatment time and better tolerability than conventional rTMS (Wu et al., 2018). Li et al. (2022) reported the positive contribution of iTBS to improved cognitive function in patients with PSCI, especially the executive and memory functions of patients. Tsai et al. (2020) further compared the efficacy of rTMS, iTBS, and sham stimulation in patients with PSCI. They reported that the 5-Hz rTMS group was significantly better than the sham stimulation group regarding the total Repeated Battery for Assessment of Neuropsychological Status (RBANS) scores, especially for attention and memory. The iTBS group also showed better improvements in the total RBANS scores and delayed memory than the sham stimulation group. However, regarding attention, the 5-Hz rTMS group showed a better reflex effect than the iTBS group. Furthermore, in the Tsai study, patients' mood disorders did not significantly improve after rTMS treatment, reinforcing the idea that rTMS treatment improves patients' cognitive function rather than because of changes in their mood disorders. Because of the lack of sufficient RCTs and uniformity in outcome indicators, we could not perform further subgroup analyses of both iTBS and conventional rTMS treatment modalities to compare the differences in their efficacy. However, the results obtained from the analyzed studies indicate that iTBS is equally effective in treating cognitive dysfunction in patients with PSCI. This finding is also well-supported by the same mechanism of action in rTMS and the positive role of iTBS in cognitive dysfunction caused by other diseases such as Parkinson's disease and Alzheimer's disease. More randomized controlled trials related to iTBS vs. conventional rTMS for cognitive dysfunction in patients with PSCI should be encouraged to inform the clinical treatment of PSCI.

In our included RCTs, the majority of rTMS stimulation sites in the intervention group were in the prefrontal cerebral cortex (DLPFC), and only one study (Fregni et al., 2006) used the M1 area as the stimulating site. Several studies (Gaudeau-Bosma et al., 2013; Parkin et al., 2015; Alcalá-Lozano et al., 2018) have shown that the DLPFC is related to the control of cognition, such as operational sequencing, attention and memory. Moreover, the DLPFC plays an important role in the central executive network, which is responsible for a high degree of control of cognitive functions, such as attention and working memory (Bressler and Menon, 2010). However, in patients with PSCI, the choice of stimulation site for rTMS when the lesion site is in the left or right cerebral hemisphere, or even bilateral cerebral hemispheres, the choice of stimulation site for the left DLPFC (L-DLPFC), or right DLPFC (R-DLPFC) or bilateral DLPFC was not uniform in these studies. Of these, 8 studies (Kim et al., 2010; Liu, 2017; Liu et al., 2020; Li et al., 2020, 2022; Tsai et al., 2020; Yin et al., 2020; Chu et al., 2022) chose L-DLPFC as the stimulation site, one study (Lu et al., 2015) chose R-DLPFC and one study selected bilateral cerebral hemispheres (Du and Wu, 2005). Although most investigators used L-DLPFC as the stimulation site, there was no evidence that this choice of stimulation site was more advantageous. In addition, because these studies were not scored using uniform outcome metrics, the studies we included were not equipped to perform subgroup analyses of this aspect of the stimulation site. However, optimal stimulation site selection and accurate localization of rTMS are key factors in improving the efficacy of rTMS treatment. Therefore, more RCTs with large sample sizes using different rTMS stimulation sites and multicenter studies are needed to further investigate the effect of different stimulation sites on the efficacy. The aim is to find the optimal stimulation site to improve the clinical efficacy of rTMS for patients with PSCI.

Adverse events reported for clinical use of rTMS include seizures, transient syncope, perceived transient hearing changes, and transient headache adverse reactions (Rossi et al., 2009; Hu et al., 2012). Only 4 of the included RCTs reported adverse reactions during rTMS treatment, comprising transient dizziness and headache that resolved with rest. No patient withdrew from the study due to adverse reactions. Therefore, rTMS treatment of patients with PSCI by professional therapists according to the treatment parameters recommended in the rTMS safety guidelines (Rossi et al., 2021) is generally safe after adequate assessment of the patient's physical condition and exclusion of contraindications.

## Limitations

There are several limitations to this meta-analysis that should be considered. First, the sample sizes of the RCTs we included were small (*n* < 100), and too small a sample size could easily bias the assessment of treatment effects and overestimate the efficacy of rTMS. Second, our included studies used different cognitive functioning rating scales, such as the MMSE, LOTCA, MoCA, and Repeated Battery for Assessment of Neuropsychological Status (RBANS), which focus on different aspects of cognitive impairment and elevate the heterogeneity between studies to some extent. Third, although our studies all showed the positive efficacy of rTMS in patients with PSCI, these studies did not report follow-ups of patients to further confirm the long-term effects of rTMS. Fourth, our included studies differed in terms of stimulation site, intensity, frequency, and the number of pulses. The number of available studies did not allow for a more detailed subgroup analysis. Therefore, in the future, more multicenter follow-up, double-blind, randomized controlled trials should be conducted to facilitate longitudinal and cross-sectional comparisons of different stimulation parameters of rTMS, to determine the optimal treatment protocol, and to improve the clinical efficacy of rTMS in patients with PSCI.

## Conclusions

The current analysis of the evidence suggests that rTMS safely and effectively promotes cognitive recovery in patients with PSCI, with a few transient adverse effects occurring during treatment, but all within the patient's tolerance range and with no significant negative effects on the patient. Both high-frequency rTMS and low-frequency rTMS stimulation of DLPFC in PSCI patients were effective in accelerating the improvement of cognitive function and enhancing the ability of patients to perform activities of daily living. There is no significant difference in the treatment effect of high-frequency rTMS and low-frequency rTMS in patients with PSCI. In addition, given the small sample size currently included, regarding rTMS for PSCI, more multicenter RCTs with large sample sizes are needed in the future to explore the selection of optimal stimulation sites and stimulation parameters to provide more effective and precise treatment for patients with PSCI.

## Data availability statement

The raw data supporting the conclusions of this article will be made available by the authors, without undue reservation.

## Author contributions

Conception and design and drafted the manuscript: CG and HH. Critical revision: ZL and HL. Collection and analysis of the data: YL, X-MP, and Y-BZ. Supervision: LX and YL. Final approval of the article: YL, Y-BZ, and M-YW. All authors have full access to all data in the study and are responsible for data completeness and accuracy of data analysis.
